# Treatment of Enuresis Nocturna in Children

**Published:** 1890-12

**Authors:** 


### TREATMENT OF ENURESIS NOCTURNA IN CHILDREN

Dr. Van Tienhoven, Director of the Krankenhaus, in Haag, advances an enlightening theory on the cause of this troublesome condition appearing in otherwise perfectly healthy children; as a result of his deductions, he recommends the following successful therapeutic measure. The bladder, performing its functions well during the day, has no power to hold the urine during the night, consequently relieves itself of it. The question now is, is it the *musc. detrusor urinæ* or the *musc. sphincter vesicæ*, or both, the one perhaps less, the other more involved? Dr. Van Tienhoven believes that the sphincter muscle is not strong enough to retain the urine that has collected during the first part of the night; that it permits the urine collected before the orifice of the urethra to pass into the *pars prostatica urethræ* and at this point, through reflex action, the *musc. detrusor* is stimulated, and under its action the entire contents of the bladder are expelled.

This conviction suggested to Dr. Van Tienhoven the idea that children suffering from enuresis should sleep with the pelvis elevated. Experiments on the cadaver prove that after elevation of the pelvis the bladder will hold a considerable amount of urine before the height of the orifice of the urethra is reached. Fourteen cases, treated according to this theory, without other therapeutic means, were cured in a short time. The necessary elevation is obtained by raising the foot of the bed to a plane of 45 degrees. The children must go to bed with an empty bladder and without having taken liquids for some time before retiring. They sleep well in the elevated position and awake without complaint of any kind.—*Correspondenz-Blatt fuer Schweizer Aerzte*, Sept., 1890.—*Pittsburg Review.*

